# Encapsulation of Docosahexaenoic Acid Oil Substantially Improves the Oxylipin Profile of Rat Tissues

**DOI:** 10.3389/fnut.2021.812119

**Published:** 2022-01-13

**Authors:** Jun Wang, Jordane Ossemond, Yann Le Gouar, Françoise Boissel, Didier Dupont, Frédérique Pédrono

**Affiliations:** ^1^French National Research Institute for Agriculture, Food and Environment (INRAE), Mixed Research Units of Science and Technology of Milk and Eggs (STLO), Rennes, France; ^2^Institut Agro, Agrocampus Ouest, Rennes, France

**Keywords:** DHA, encapsulation, oxylipin, brain, heart, rat

## Abstract

Docosahexaenoic acid (DHA) is a major n-3 polyunsaturated fatty acid (PUFA) particularly involved in cognitive and cardiovascular functions. Due to the high unsaturation index, its dietary intake form has been considered to improve oxidation status and to favor bioaccessibility and bioavailability as well. This study aimed at investigating the effect of DHA encapsulated with natural whey protein. DHA was dietary provided as triacylglycerols to achieve 2.3% over total fatty acids. It was daily supplied to weanling rats for four weeks in omelet as food matrix, consecutively to a 6-hour fasting. First, when DHA oil was encapsulated, consumption of chow diet was enhanced leading to promote animal growth. Second, the brain exhibited a high accretion of 22.8% DHA, which was not improved by dietary supplementation of DHA. Encapsulation of DHA oil did not greatly affect the fatty acid proportions in tissues, but remarkably modified the profile of oxidized metabolites of fatty acids in plasma, heart, and even brain. Specific oxylipins derived from DHA were upgraded, such as Protectin Dx in heart and 14-HDoHE in brain, whereas those generated from n-6 PUFAs were mainly mitigated. This effect did not result from oxylipins measured in DHA oil since DHA and EPA derivatives were undetected after food processing. Collectively, these data suggested that dietary encapsulation of DHA oil triggered a more efficient absorption of DHA, the metabolism of which was enhanced more than its own accretion in our experimental conditions. Incorporating DHA oil in functional food may finally improve the global health status by generating precursors of protectins and maresins.

## Introduction

Docosahexaenoic acid (DHA, 22:6n-3) is an essential n-3 polyunsaturated fatty acid (PUFA), mainly known for its health benefits on brain and heart. This bioactive compound presents a large range of beneficial outcomes inherent to the modulation of both membrane structure and cell functions. Therefore, DHA improves neurological activities by enhancing neurogenesis and synaptic plasticity, or by reducing neuroinflammation (1). Its high body level is related to the lower risk of developing cardiovascular disease (2, 3) and myocardial infarction as well (4). The functionality of DHA is likely to be mostly implemented through the synthesis of derivatives such as oxidized metabolites of FA (oxylipins) or endocannabinoid-like mediators. These derivatives may be generated either from DHA itself or from other FA, such as arachidonic acid (ARA, 20:4n-6), dihomo-α-linolenic acid (DGLA, 20:3n-6), linoleic acid (LA, 18:2n-6), and eicosapentaenoic acid (EPA, 20:5n-3). DHA accumulates, indeed, in cell membranes concomitantly with the reduction of n-6 FA, principally ARA, or is retroconverted into EPA. Therefore, the effect of DHA may be attributed both to the rise of its own derivatives and the decline of derivatives synthesized from other FA. Many studies have reviewed the importance of the dietary supply of DHA as a strategy to increase tissue DHA concentrations (5–7). Although modulating the FA content in brain still represents a challenge comparatively to other tissues, dietary interventions may be effective in targeting serum lipid pools important for brain DHA uptake (8). Thereby, several works have shown a higher (9–11) or equal (12–14) bioavailability of DHA esterified to phospholipids (PL) in comparison to DHA esterified to triacylglycerols (TAG). Other works are more controversial (15, 16), insomuch as a higher concentration of circulating DHA would not predict brain accretion of DHA (17–19). Furthermore, dietary supplementation of DHA was shown to generate higher levels of its oxygenated derivatives as oxylipins (20, 21). These lipid mediators are synthesized from PUFA by cyclooxygenase, lipoxygenase, or cytochrome P450 ω-hydroxylase or epoxygenase enzymes, or by non-enzymatic autoxidation (22). The oxylipin pattern depends both on the dietary intake of n-6 or n-3 PUFA, and on the endogenous synthesis of long-chain PUFA considering enzyme competition between n-6 and n-3 families in tissues. The structural diversity of oxylipins is notably high for most of the PUFA and foreshadows a diversity of biological functions as well. Hence, these essential signaling messengers are usually involved in physiological responses to maintain homeostasis, contain infection or restrain inflammation process by acting through specific membrane receptors (23, 24). More particularly, oxygenation of DHA forms docosanoids, including specialized pro-resolving mediators (SPM) such as maresins, D-resolvins, and protectins (25). The literature reports their bioactive implying on pathophysiological process as they improve immune response, resolution of neuro-inflammation, or other metabolic disorders including diabetes, atherosclerosis, non-alcoholic fatty liver disease, or hepatic steatosis (26–30). These pleiotropic properties justify the interest for DHA, and its food intake other than based on PL or TAG esterification has to be considered as well.

An alternative approach to optimize dietary DHA intake is the use of functional food based on encapsulation. The food-grade delivery system was originally developed to protect bioactive ingredients notably from degradation such as oxidation. Many techniques using different encapsulant materials were processed in diverse food matrices for this purpose (31). Nevertheless, controlling the colloidal state of lipophilic molecules underlies the control of composition, size, and surface characteristics of particles, to ensure the required bioavailability. Pickering emulsification of food oil has recently received substantial interest as it relies more specifically on adsorption of bioparticles rather than synthetic surfactants to the oil-water interface, which confers resistance to droplet coalescence and improves the stability of emulsion (32–34). In this study, Pickering emulsion was prepared with natural whey protein to encapsulate DHA enriched-TAG, as previously described (35). Although fermented milk products are widely used as a vector of encapsulated nutrients, our previous study showed that eggs present an interesting bioavailability of DHA according to the recipe (omelet, hard-boiled egg, or mousse) (36). In the present study, DHA was therefore encapsulated in heat-denatured whey proteins. Whey protein isolate is commonly used in food industry due to a variety of functionalities such as emulsification or gelation properties. The heat-denatured form was favored in this study considering the cooking process that needed to be applied during the food manufacture. For this reason, whey protein-based microcapsules of DHA oil were incorporated into eggs and baked thereafter as omelets. Then, the rats were daily fed with DHA enriched-omelet for four weeks. The effect of encapsulation was finally assessed on DHA accretion, specifically in brain and heart, and subsequently on FA derivative formation such as oxylipins.

## Materials and Methods

### Animal Experiment

**Figure 1:**
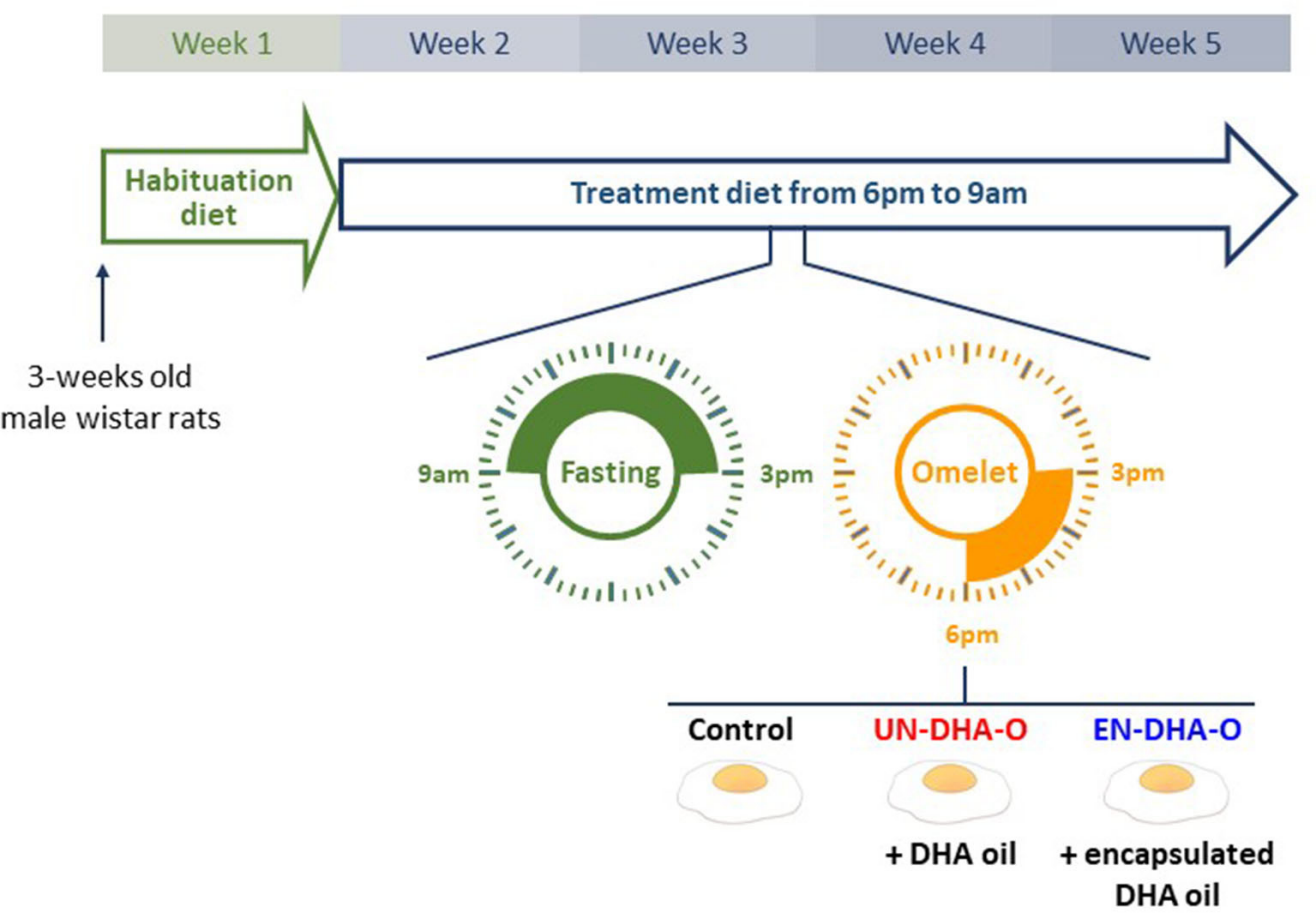
The experimental design. Rats were acclimated with the habituation diet for one week. They were then fed with the treatment diet (T-diet) for four weeks. During this period, the rats were daily fasted for 6 hours before receiving 3g of omelet containing DHA as oil (UN-DHA-O) or as encapsulated oil (EN-DHA-O).

All handling protocols performed complied with the European Union Guideline for animal care and use (2010/63/CEE; decree 2013-118). The present project was authorized by the French Ministry of Higher Education and Research under the number 27678-2020100615388160 v3. Male Wistar rats (3 weeks-old) were supplied by the Janvier Labs Breeding Center (Le Genest-Saint-Isle, France). The animal experiment was performed at ARCHE Biosit (University of Rennes I, Campus of Villejean, Rennes, France). They were randomly divided into 3 groups of 8 animals and housed in pairs. They were acclimated for one week with the habituation diet (H-diet) and were then fed with the treatment diet (T-diet) for four weeks (Figure 1). During this period, the rats were daily fasted from 9 a.m. to 3 p.m. Each group then received 3g per rat of omelet containing no DHA, DHA oil, or encapsulated-DHA oil. Thereby, the rats were separated for three hours by a plexiglass plate positioned in the middle of the cage, allowing individual consumption of omelet serving while guaranteeing eye and smell contact between congeners. At 6 p.m., the T-diet was restored until the next morning. The animals had access to water *ad libitum*. Rodent chows (H-diet and T-diet) were distributed *ad libitum* as well at the indicated time slots. After four weeks of experiment, the fasted rats were anesthetized with intraperitoneal injections of ketamine (100 mg/kg, Imalgene®1000, Mérial, Lyon, France) and xylazine (10mg/kg, Rompun® 2%, Bayer Animal Health, Puteaux, France). Blood was collected in heparin tubes by cardiac puncture. Plasma was separated from red blood cells (RBC) by centrifugation (2000 rpm, 15 min, 15 °C). Eyes were sampled in phosphate buffer saline whereas liver, heart, and brain (frontal cortex was studied here) were snap-frozen and stored at −80 °C until analysis.

### Diet

Diets were rodent chows prepared according to the AIN-93-G formulation and made at the Unité de Préparation des Aliments Expérimentaux of INRAE (Jouy en Josas, France) (37). The H-diet was composed of 20.6% proteins, 64.8% carbohydrates, and 5% lipids. The T-diet was composed of 20.8% proteins, 65.1% carbohydrates, and 4.5% lipids. Both diets contained 5.1% fibers and 4.5% mineral and vitamin mix (Table 1) (38). Pellets were formulated by adding a mixture of vegetable oils specific for each diet (Table 1), considering the consumption of eggs during the 4-week period of treatment.

**Table 1:** The composition of diets **(A)** and oil mix **(B)**.

**%**	**H-diet**	**T-diet**
**A**		
Starch	54.6	54.9
Sucrose	10.2	10.2
Cellulose	5.1	5.1
Casein	20.6	20.8
Oil mix	5.0	4.5
Mineral mix	3.5	3.5
Vitamin mix	1.0	1.0
**B**		
Linseed	1.4	2.9
Sunflower	8.6	8.8
Olive	13.5	12.6
Rapeseed	25.0	25.2
Palm	51.5	50.5

*The composition of dry diets based on the AIN-93-G is presented for the habituation and the treatment diets. Both diets contained a mixture of vegetable oils formulated according to the daily consumption of eggs*.

The T-diet was designed similarly to the H-diet to achieve the same final FA profile per day. Food consumption was previously estimated at 20 g per day of rodent chow combined with 3 g omelet serving over this growth period. In that condition, egg was supposed to contribute to the height of 0.5% lipids and T-diet of 4.5% lipids. The mixture of vegetable oils was adjusted in T-diet as a consequence in considering the FA profile of egg. The portion of linseed was thus increased between H-diet and T-diet to compensate the low level of 18:3n-3 (α-linolenic acid, ALA) in omelet, in order to maintain the ratio n-6/n-3 to 5 with 3.4% of 18:3n-3 in average.

### DHA Encapsulation and Omelets

DHA oil was prepared enzymatically from fish oil and was composed of DHA-enriched TAG containing 615 μg/mg DHA, 31 μg/mg docosapentaenoic acid n-3 (DPAn-3, 22:5n-3), and 36 μg/mg EPA, accounting for 76.5, 3.9, and 4.5% of total FA, respectively (Polaris, Quimper, France). DHA oil was daily encapsulated with heat-denatured whey protein isolate as described previously (35). The supplementation of proteins was therefore 300 μg per day, corresponding to less than 0.1% of proteins from eggs.

The omelet was daily prepared with a whole egg (Moisan aviculture, Plestan, France) homogenized by ultraturrax (10 000 rpm, 30 sec). Then, encapsulated-DHA oil (EN-DHA-O), heat-denatured whey protein isolate dispersion alone (Control) or completed with the DHA oil (UN-DHA-O) were added and mixed by stirring (500 rpm, 5 min). The eggs were finally molded and cooked in a water bath (80°C, 10 min).

### Fatty Acid and Dimethylacetal Analysis

The lipids from omelets and tissues were extracted according to the Folch's method (39). DHA oil, vegetable oils, and lipids from omelets and tissues were saponified with 0.5 mol/L NaOH in methanol at 70°C for 20 min and methylated with BF_3_ (14 % in methanol) at 70°C for 15 min (Supplementary Figure 1). Fatty acid methyl esters (FAMEs) and dimethylacetals (DMAs) were extracted with pentane and then separated by a QP 2010-SE gas chromatograph coupled to a mass spectrometer (Shimadzu, Marne-La-Vallée, France) equipped with a BPX70 capillary column (120 m, 0.25 mm i.d., 0.25 μm film, SGE Trajan, Chromoptic, Paris). Helium was used as carrier gas at a constant velocity of 27,5 cm/sec. The temperature of injector was adjusted to 250°C. The column temperature ramped from 50 to 175°C at 20 °C/min and then from 175 to 240°C at 2 °C/min. The mass spectrometer was operated under electron ionization at 0.2 keV and 200 °C source temperature. Analyses were performed in scan mode over the m/z range of 30–450 amu. Components were identified by using the National Institute of Standards and Technology (NIST) mass spectral library (version 2.01) in addition to commercial standards. Concentrations were determined by using 17:0 as the internal standard and were calculated by standard curves of either FAME or DMA. Results were expressed as a mass percentage of the total FA or total DMA, and as concentrations for total FA and total DMA as well.

### Oxylipin Analysis

Oxidized metabolites of PUFA were quantified in DHA oil, omelets as well as plasma, heart, and brain by liquid chromatography combined with tandem mass (LC-QQQ) by the MetaToul lipidomic core facility (Justine Bertrand-Michel and Pauline Le Faouder, I2MC, Inserm 1048, MetaboHUB-ANR-11-INBS-0010 Toulouse, France). Briefly, frozen tissues (250 mg) were crushed with a FastPrep ®-24 Instrument (MP Biomedical, Illkirch-Graffenstaden, France) in 500 μL of Hank's balanced salt solution (Thermo Fisher Scientific, Illkirch-Graffenstaden, France). After 2 crush cycles (6.5 m/sec, 30 sec), cold methanol (300μL) and internal standard (5 μL of LXA4-d5, LTB4-d4 and 5HETE-d8) were added to homogenates. After centrifugation (15 min at 2000 g at 4°C), supernatants were diluted in 2 mL H_2_O and then submitted to solid phase extraction using OASIS HLB 96-well plate (30 mg/well, Waters, Saint-Quentin-en-Yvelines, France) pretreated with methanol (1 mL), equilibrated with 10% methanol (1 mL), and washed after sample application with 10% methanol (1 mL). Lipid mediators were finally eluted with 1 mL of methanol and reconstituted in 10 μL methanol prior to LC-QQQ analysis (40). They were separated on a ZorBAX SB-C18 column (50 mm, 2.1 mm i.d., 1.8 μm film) using Agilent 1,290 Infinity HPLC system coupled to an ESI-triple quadruple G6460 mass spectrometer (Agilent Technologies, Les Ulis, France). Data were acquired in multiple reactions monitoring mode with optimized conditions (ion optics and collision energy). Peak detection, integration, and quantitative analysis were done using Mass Hunter Quantitative analysis software (Agilent Technologies) based on calibration lines built with eicosanoid standards (Interchim, Montluçon, France).

### Statistics

The results are expressed as the mean ± SEM of 8 animal samples per group. Data analysis was performed using R software. Correlations were evaluated by Pearson correlation coefficients and the analysis of variance was done by ANOVA followed by a *post hoc* test depending on the normality of the data distribution. The significance of the effect observed with the DHA oil or the encapsulated-DHA oil was marked by an asterisk or different letters when *p* < 0.05.

## Results

### Diet Characteristics

The animals were accommodated with the H-diet for one week. This rodent chow was composed of monoenes (18:1n-9) and saturates (16:0 and 18:0) as major FA, and contained 17.0% LA (18:2n-6) and 3.4% ALA corresponding to a ratio of 5 (Table 2). This composition contrasts with the breeding rodent chow, which classically contains 50% LA and 9% ALA. The composition of the T-diet was close to the H-diet but was reduced in lipid content considering the daily supply of eggs. Specifically, ALA was increased (4.3% of the total FA) to achieve a n-6/n-3 ratio of 4 in the T-diet since the supply of omelet reduces the n-3 FA content (only 0.4–0.5% of 18:3n-3). Overall, the FA profile of omelets was closed to the FA profile of rodent chow. LA and ALA were however lower in omelets as compared to the H-diet and T-diet, balanced by a greater proportion of 18:0. The T-diet was designed according to the FA profile of omelets, considering the expected daily consumption of both omelet and rodent chow. After one week of acclimation with H-diet, the T-diet was distributed during the 4-week treatment with eggs. Each group of animals received 3g omelet per day, the supplementation of which differed according to the input form of DHA oil. DHA was integrated to the recipe to reach 10% of the total FA of omelet, while only 0.8% of endogenous DHA was measured from control eggs (Table 2).

**Table 2:** The fatty acid composition of the diets and the omelets.

**%**	**Diets**		**Omelets**		
	**H-diet**	**T-diet**	**Control**	**UN-DHA-O**	**EN-DHA-O**
12:0	0.1	0.1	0.0	0.0	0.0
14:0	0.6	0.6	0.3	0.3	0.3
16:0	26.2	25.7	25.3	22.5	22.1
18:0	3.4	3.4	8.0	7.1	7.2
20:0	0.4	0.4	0.0	0.0	0.0
22:0	0.2	0.2	0.0	0.0	0.0
24:0	0.1	0.1	0.0	0.0	0.0
**Saturates**	**31.0**	**30.5**	**33.6**	**29.9**	**29.6**
14:1n-5	0.0	0.0	0.1	0.0	0.1
**n-5**	**0.0**	**0.0**	**0.1**	**0.0**	**0.1**
16:1n-7	0.2	0.2	2.9	2.6	2.6
18:1n-7	1.4	1.4	2.0	1.8	1.8
**n-7**	**1.6**	**1.6**	**4.9**	**4.4**	**4.4**
16:1n-9	0.0	0.0	0.7	0.6	0.6
18:1n-9	46.2	45.7	42.2	37.9	37.2
20:1n-9	0.5	0.4	0.1	0.2	0.3
22:1n-9	0.3	0.3	0.0	0.0	0.0
24:1n-9	0.0	0.0	0.0	0.2	0.2
**n-9**	**47.0**	**46.4**	**43.0**	**38.9**	**38.3**
18:2n-6	17.0	17.2	14.8	13.0	12.9
20:4n-6	0.0	0.0	2.0	1.9	1.9
22:4n-6	0.0	0.0	0.0	0.1	0.1
22:5n-6	0.0	0.0	0.3	0.6	0.6
**n-6**	**17.0**	**17.2**	**17.1**	**15.6**	**15.5**
18:3n-3	3.4	4.3	0.5	0.4	0.5
20:5n-3	0.0	0.0	0.0	0.5	0.5
22:5n-3	0.0	0.0	0.0	0.5	0.5
22:6n-3	0.0	0.0	0.8	9.8	10.6
**n-3**	**3.4**	**4.3**	**1.3**	**11.2**	**12.1**

*Lipids from croquettes and omelets were extracted according to the Folch's method. The fatty acid profile performed by GC-MS was determined for the habituation and the treatment diets, and the different omelets prepared with unencapsulated-DHA oil or encapsulated-DHA oil*.

*Bold values correspond to the sum of fatty acids per family*.

*Colored values correspond to the type of omelets: black for control, red for UN-DHA-O and blue for EN-DHA-O*.

### Food Intake and Animal Growth

**Figure 2:**
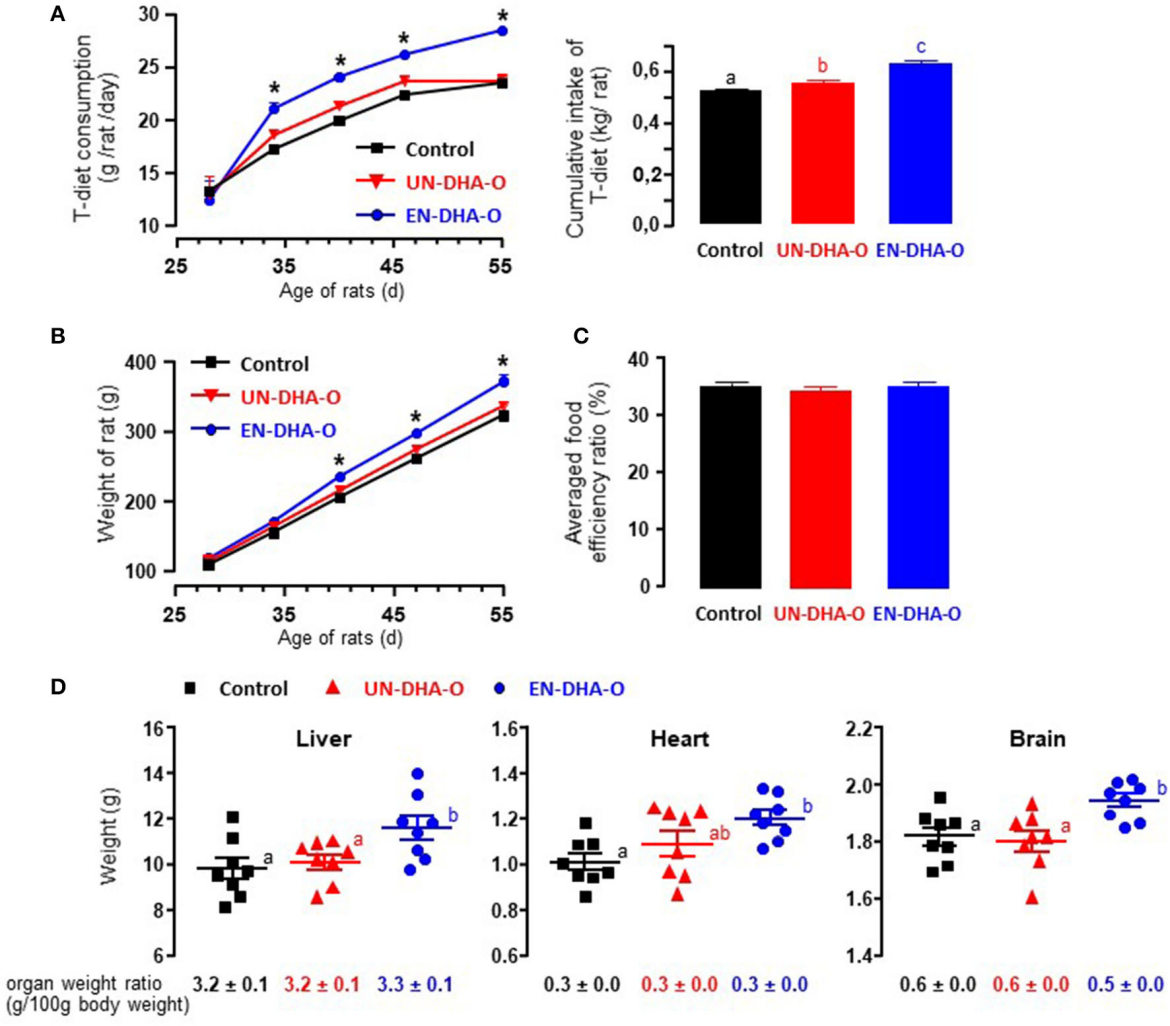
The food intake **(A)**, the growth **(B)**, the food efficiency ratio **(C)**, and the tissue weights **(D)** of rats. Food consumption of T-diet and animal growth were followed during the four-week experiment. Food efficiency ratio averaged over the four-week experiment was calculated with the daily consumption of T-diet and omelets over the weight gain of growing animals. The effect of diets was determined as well on the tissue weight prior to lipid analysis. a, b, c and ab indicate significant differences between the three groups when p < 0.05; ab means no difference with a and no difference with b. ^*^indicates significant differences between EN-DHA-O and both Control and UN-DHA-O when p < 0.05.

The animals were fasted for 6 h before receiving individually the portion of omelets, the consumption of which was complete for each group of rats during the entire experiment. The T-diet intake was followed as well and a significant increase in the food consumption was observed from the first week with EN-DHA-O (Figure 2A). More precisely, the cumulative intake of the T-diet over the 4-week experiment was increased by 6% with UN-DHA-O and by 20% with EN-DHA-O compared with the control. This behavior was coupled with the animal growth since the final weight of rats fed EN-DHA-O was significantly higher by 15% than the control group (Figure 2B). Food efficiency ratio, calculated by the ratio between body weight gain and food intake (T-diet and omelet), was not different between groups and averaged 38.4, 37.9, and 39.3% for Control, UN-DHA-O, and EN-DHA-O respectively, over the four-week experiment (Figure 2C). Likewise, the weights of organs were also significantly augmented in the EN-DHA-O group by 24% for liver, by 19% for heart, and by 6% for brain as compared with the control group (Figure 2D). Nevertheless, when the organ weights were normalized with body weights of animals, no significant differences were obtained between groups, which subtends that the raise in organ weights resulted from rat growth. The weight gain of animals resulted presumably from an increased energy intake, since the FA proportion was similar between groups. The FA profile was indeed estimated in the view of the measured consumption of the T-diet per group on the one hand, and the complementary omelet on the other (Table 3). The FA profile combining T-diet and omelet was similar to the FA profile of the H-diet and no major difference in FA proportions or n-6/n-3 ratio was observed between groups. In addition, both diets enriched in DHA oil accounted for a supplementation of TAG-DHA of 2.3%, since the FA was supplied equivalently between the groups through the distribution of 3g omelet per day.

**Table 3:** The fatty acid composition of the daily intake of T-diet and omelets.

**%**	**T-diet with different omelets**		
	**Control**	**UN-DHA-O**	**EN-DHA-O**
12:0	0.1	0.1	0.1
14:0	0.5	0.5	0.5
16:0	25.6	25.4	25.4
18:0	4.5	4.4	4.4
20:0	0.3	0.3	0.3
22:0	0.1	0.1	0.1
24:0	0.1	0.1	0.1
**Saturates**	**31.2**	**30.9**	**30.9**
16:1n-7	0.8	0.8	0.8
18:1n-7	1.6	1.5	1.5
**n-7**	**2.4**	**2.3**	**2.3**
16:1n-9	0.2	0.2	0.2
18:1n-9	44.8	44.8	44.8
20:1n-9	0.4	0.4	0.4
22:1n-9	0.2	0.2	0.2
24:1n-9	0.0	0.1	0.1
**n-9**	**45.6**	**45.7**	**45.7**
18:2n-6	16.6	16.6	16.6
20:4n-6	0.5	0.5	0.4
22:5n-6	0.1	0.2	0.2
**n-6**	**17.2**	**17.3**	**17.2**
18:3n-3	3.4	3.4	3.5
20:5n-3	0.0	0.1	0.1
22:5n-3	0.0	0.1	0.1
22:6n-3	0.2	2.5	2.5
**n-3**	**3.6**	**6.1**	**6.2**
n-6/n-3	4.9	4.9	4.8
**Total**	**100.0**	**102.3**	**102.3**
		TAG-DHA + 2.3%	

*The fatty acid profile was calculated per group of omelets on the base of the treatment diet consumption, and was averaged per day on the four-week experimental period. This profile corresponds to the combination between FA consumed from the T-diet and FA supplied by the 3g omelet*.

*Bold values correspond to the sum of fatty acids per family*.

*Colored values correspond to the type of omelets: black for control, red for UN-DHA-O and blue for EN-DHA-O*.

The first ascertainment showed an effect of the EN-DHA-O diet on food intake and rat growth, so we further investigated the effect of encapsulation of DHA on lipid metabolism.

### Effect of Diets on the Metabolism of FA

**Figure 3:**
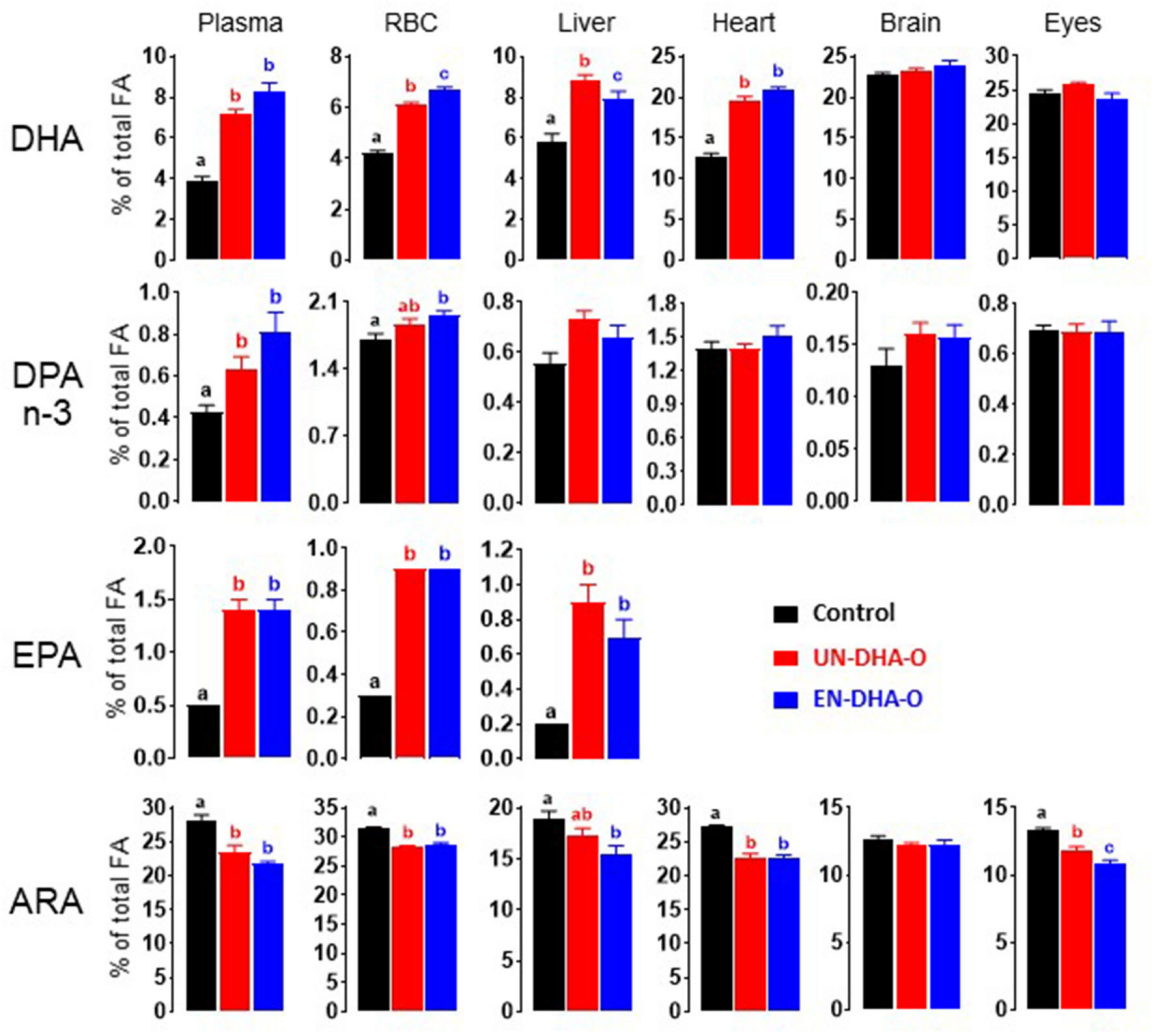
The effect of form of the DHA intake on the FA profile of rat tissues after 4 weeks of treatment. Lipids from rat tissues were extracted according to the Folch's method and the fatty acid profile was then determined by gas chromatography-mass spectrometry (GC-MS). The main results on n-3 PUFA and arachidonic acid are presented. a, b, c and ab indicate significant differences between the three groups when *p* < 0.05; ab means no difference with a and no difference with b.

The FA metabolism and particularly the accretion of DHA in tissues were then explored to assess the importance of encapsulation of the DHA oil in the diet. The analysis focused on blood, liver, heart, brain, and eyes. First, the diet supplementation of DHA induced a significant increase in DHA in tissues, except for the brain and the eyes (Figure 3). Encapsulation of the DHA oil globally tends to increase the DHA accretion in blood and heart (Supplementary Tables 1–3). In liver, a decrease in the DHA proportion was observed with EN-DHA-O, but coupled with a slight increase in the FA content, the DHA concentration was not changed. The impact of the diets was also estimated on precursors of DHA, minorly present in the DHA oil. Thus, EPA was significantly increased by the DHA supplementation in plasma, RBC, and liver without any significant effect of the encapsulation of the DHA oil. The same result was observed with DPA n-3 only in plasma (Figure 3). Second, the proportion of n-6 FA was significantly decreased with the DHA-enriched diets in all tissues, depending on the considered FA. The main effect was obtained in heart on the entire n-6 family (Supplementary Table 2) and in eyes especially on ARA (Supplementary Table 3). Only the eyes exhibited a significant effect of the encapsulation of the DHA oil by slightly reducing ARA from 11.8% in UN-DHA-O to 10.9% in EN-DHA-O. Otherwise, no major effect of the DHA supplementation was observed on the FA profiles. Analyses were further performed on particular lipids or derivatives.

### Effect of Diets on the DMA Profile

The brain and heart are particularly rich in plasmalogens, which are specific etherlipids known as reservoirs of polyunsaturated FA. DMA compounds are derived from the aldehyde chain of plasmalogens present at the *sn*-1 position of the glycerol and were synthesized through the lipid saponification and methylation steps. They co-eluted with FAME. The DMA profile was thus analyzed to quantify plasmalogens (Table 4). Therefore, the DMAs were only measured in brain, heart, RBC, and eyes. The brain contained an average of 2 μg of DMA per mg of tissues, against 0.4μg/mg in heart and 0.1μg/mg in RBC and eyes. Moreover, the alkenyl chain was discriminant between tissues. For instance, in brain, the major DMA was composed of 18:0 (53% in all groups), then 16:0 (22%), 18:1n-9 (15%), and 18:1n-7 (10%). The profile of DMA was completely different in heart with 63% of 16:0, then 18:1n-9 (23%) and 18:0 (13%). Nonetheless, no effect of the diet was measured on the DMA profile. Surprisingly in eyes, the DMA content was reduced by half with the DHA supplementation passing from 0.12μg/mg to 0.06μg/mg and 0.07μg/mg with the UN-DHA-O and EN-DHA-O diets, respectively. Moreover, a switch in the DMA profile was observed in favoring 16:0-DMA (+13.9% with UN-DHA-O and +8.3% EN-DHA-O) to the detriment of 18:1-DMA, but without impacting the 18:0-DMA. No change of the DMA profile was, however, observed by encapsulation of the DHA oil.

**Table 4:** The dimethylacetal profile of RBC, heart, brain, and eyes.

	**%**	**16:0-** **DMA**	**18:0-** **DMA**	**18:1n-9-** **DMA**	**18:1n-7-** **DMA**	**μg/mg**
**RBC**	**Control**	29.2 ± 0.3	33.2 ± 0.3	31.6 ± 0.3	6.0 ± 0.1	0.12 ± 0.00
	**UN-DHA-O**	30.3 ± 0.3	32.6 ± 0.4	31.8 ± 0.5	5.3 ± 0.2	0.12 ± 0.00
	**EN-DHA-O**	30.0 ± 0.5	33.0 ± 0.4	31.6 ± 0.7	5.4 ± 0.3	0.13 ± 0.00
**Heart**	**Control**	63.2 ± 1.0	13.4 ± 1.0	23.4 ± 1.2	0.0 ± 0.0	0.38 ± 0.01
	**UN-DHA-O**	64.2 ± 1.8	13.6 ± 1.5	22.2 ± 1.4	0.0 ± 0.0	0.36 ± 0.02
	**EN-DHA-O**	62.2 ± 1.3	15.1 ± 0.7	22.7 ± 1.5	0.0 ± 0.0	0.42 ± 0.02
**Brain**	**Control**	22.2 ± 0.9	52.6 ± 0.7	14.9 ± 0.4	10.3 ± 0.3	2.17 ± 0.07
	**UN-DHA-O**	21.5 ± 0.7	51.7 ± 0.6	16.3 ± 0.7	10.5 ± 0.4	2.49 ± 0.13
	**EN-DHA-O**	21.6 ± 0.5	52.7 ± 0.6	15.4 ± 0.6	10.3 ± 0.4	2.30 ± 0.11
**Eyes**	**Control**	26.8 ± 0.3^a^	34.3 ± 0.4	20.0 ± 0.4^a^	18.9 ± 0.2^a^	0.12 ± 0.00^a^
	**UN-DHA-O**	40.7 ± 2.1^b^	34.8 ± 3.0	11.1 ± 2.4^b^	13.4 ± 2.2^b^	0.06 ± 0.00^b^
	**EN-DHA-O**	35.1 ± 1.3^b^	34.8 ± 0.6	15.9 ± 0.8^ab^	14.2 ± 1.0^ab^	0.07 ± 0.01^b^

*Lipids of tissues were extracted by the Folch's method and the dimethylacetal profile was determined by GC-MS*.

a, b, c and ab *indicate significant differences between the three groups when p < 0.05; ab means no difference with a and no difference with b*.

*Colored values correspond to the type of omelets: black for control, red for UN-DHA-O and blue for EN-DHA-O*.

### Effect of Diets on Oxidized Metabolites of PUFA

Considering the slight effect of diets on the FA profiles in tissues, we further investigated the oxidized metabolites. First of all, oxylipins were quantified in DHA oil and food matrices as well (Table 5). Eight oxidized metabolites from n-6 and n-3 PUFA were measured, mainly from LA and DHA. Hence, DHA oil contained a majority of 13-HODE and 9-HODE derived from LA, followed by 17-HDoHE and 14-HDoHE derived from DHA. Much less present, few oxylipins of ARA and 18-HEPE from EPA were detected as well. When oxylipins were then measured in omelets, 13-HODE, 9-HODE, and 5-HETE were the only oxidized metabolites quantified in all groups, even in DHA-enriched omelets, where the DHA oil was added. No oxylipins from DHA and EPA were detected in UN-DHA-O and EN-DHA-O, whereas a small amount of these compounds was expected considering the supplementation with the DHA oil.

**Table 5:** The oxidized derivatives of fatty acids from DHA oil and omelets.

			**Omelets (ng/3 g omelet)**			
**FA**		**DHA oil (ng/mL)**	**Control**	**UN-DHA-O**	**EN-DHA-O**	Expected^*^
LA	13-HODE	36.61	6.61	4.03	6.15	1.81
	9-HODE	31.27	6.20	2.92	4.78	1.55
ARA	15-dPGJ_2_	0.52	0.00	0.00	0.00	0.03
	15-HETE	Trace	Trace	Trace	Trace	Trace
	5-HETE	0.94	2.16	1.19	1.53	0.05
EPA	18-HEPE	2.21	0.00	0.00	0.00	0.1
DHA	17-HDoHE	17.57	0.00	0.00	0.00	0.87
	14-HDoHE	13.07	0.00	0.00	0.00	0.65

*The oxidized metabolites of fatty acids were quantified by LC-QQQ as mentioned in methods on DHA oil used to prepare omelets*.

* *A column named “expected” results from calculations on oxylipin concentrations expected after supplementation of egg with DHA oil, considering the oxylipin pattern from DHA oil*.

*Colored values correspond to the type of omelets: black for control, red for UN-DHA-O and blue for EN-DHA-O*.

We further analyzed the oxylipin pattern of animal tissues after the 4-week experiment. First, in plasma, three oxylipins were measured and derived from DHA but also from ARA and LA (Table 6). Overall, the supplementation of DHA strongly modulated the concentrations of oxylipins, and the encapsulation of the DHA oil greatly emphasized this effect. More specifically, 14-HDoHE derived from DHA was reduced by half with UN-DHA-O and by 6 with EN-DHA-O, as 14-HDoHE was negatively correlated with the DHA proportions in plasma (Supplementary Figure 2). In parallel, 17-HDoHE was not affected by the diet, as well as with 9-HODE from LA, whereas 13-HODE was reduced by the DHA supplementation without any influence of its encapsulation. Last, the greatest effect was the decrease in ARA derivatives by 76% with UN-DHA-O and by 93% with EN-DHA-O as compared with the control. Some of the oxylipins even became undetectable with the DHA encapsulation such as PGE_2_ or 8-HETE.

**Table 6:** The oxidized derivatives of fatty acids from plasma.

**ng/mL of plasma**		**Control**	**UN-DHA-O**	**EN-DHA-O**
LA	13-HODE	13.47 ± 1.90^a^	8.04 ± 1.01^b^	7.29 ± 1.21^b^
	9-HODE	2.56 ± 0.30	1.71 ± 0.13	1.72 ± 0.25
ARA	PGF_2α_	0.39 ± 0.09^a^	0.00 ± 0.00^b^	0.00 ± 0.00^b^
	PGE_2_	0.78 ± 0.16^a^	0.19 ± 0.02^b^	0.00 ± 0.00^c^
	PGD_2_	0.59 ± 0.21^a^	0.00 ± 0.00^b^	0.00 ± 0.00^b^
	TXB_2_	7.88 ± 2.76^a^	1.59 ± 0.41^b^	0.77 ± 0.08^c^
	15-HETE	2.98 ± 0.51^a^	1.15 ± 0.12^b^	0.64 ± 0.08^c^
	8-HETE	2.03 ± 0.33^a^	0.54 ± 0.13^b^	0.00 ± 0.00^c^
	12-HETE	384.28 ± 76.32^a^	90.73 ± 22.97^b^	25.05 ± 3.25^c^
	5-HETE	4.48 ± 0.28^a^	2.30 ± 0.14^b^	1.53 ± 0.13^c^
DHA	14-HDoHE	42.30 ± 7.53^a^	20.41 ± 4.20^b^	6.82 ± 0.87^c^
	17-HDoHE	3.70 ± 0.73	2.91 ± 0.36	2.06 ± 0.26

*The oxidized metabolites of fatty acids were quantified in plasma by LC-QQQ as mentioned in methods*.

a, b and c *indicate significant differences between the three groups when p < 0.05*.

*Colored values correspond to the type of omelets: black for control, red for UN-DHA-O and blue for EN-DHA-O*.

The analyses were then completed by oxidized metabolites from heart (Table 7). Oxylipins were altogether more numerous but quantitatively less important than in plasma. No significant effect of the diets was observed this time on 14- and 17-HDoHE derived from DHA, but another derivative named PDx was remarkably increased with the DHA encapsulation. Absent in the control, PDx reached 2.1 pg/mg with UN-DHA-O and was multiplied by 8 with EN-DHA-O. The same applied to 18-HEPE derived from EPA, since it was absent in the control and the UN-DHA-O groups but reached 20.7 pg/mg with EN-DHA-O. In contrast, the cardiac concentrations of eicosanoids derived from ARA were globally decreased with the DHA supplementation, and particularly with the DHA encapsulation for PGF_2α_ and PGE_2_. Finally, the most remarkable effect was achieved by an eicosanoid from DGLA termed 6keto-PGF_1α_, which was reduced by half with UN-DHA-O and by five with EN-DHA-O.

**Table 7:** The oxidized derivatives of fatty acids from heart.

**pg/mg protein**		**Control**	**UN-DHA-O**	**EN-DHA-O**
LA	13-HODE	772.1 ± 93.6	709.4 ± 64.9	765.3 ± 58.5
	9-HODE	220.4 ± 28.9	223.6 ± 17.7	248.3 ± 20.0
DGLA	6kPGF_1α_	437.7 ± 96.6^a^	194.6 ± 28.1^b^	88.1 ± 6.7^c^
ARA	PGF_2α_	118.1 ± 20.8^a^	51.8 ± 8.8^b^	23.6 ± 3.9^c^
	PGE_2_	82.2 ± 11.6^a^	39.1 ± 7.2^b^	24.0 ± 2.3^c^
	PGD_2_	42.8 ± 6.4^a^	20.0 ± 3.1^b^	22.5 ± 3.4^b^
	TXB_2_	94.8 ± 18.5	55.3 ± 7.4	44.5 ± 6.1
	15-HETE	494.2 ± 50.6^a^	270.8 ± 22.0^b^	313.6 ± 30.9^b^
	8-HETE	109.4 ± 14.8	88.6 ± 7.9	95.9 ± 10.7
	12-HETE	3,562.0 ± 441.1	3,213.5 ± 926.4	4,335.8 ± 536.1
	5-HETE	532.0 ± 66.0^a^	325.7 ± 19.1^b^	274.6 ± 18.2^b^
	14,15-EET	55.6 ± 11.4^a^	0.0 ± 0.0^b^	0.0 ± 0.0^b^
	5-oxo-ETE	514.4 ± 60.7^a^	306.5 ± 18.9^b^	222.5 ± 14.8^b^
	11,12-EET	102.2 ± 14.8^a^	68.9 ± 5.2^ab^	48.0 ± 6.3^b^
	8,9-EET	179.3 ± 37.2	110.9 ± 10.9	78.0 ± 9.4
	5,6-EET	248.3 ± 59.7^a^	121.1 ± 18.4^ab^	70.9 ± 16.6^b^
EPA	18-HEPE	0.0 ± 0.0^a^	0.0 ± 0.0^a^	20.7 ± 3.6^b^
DHA	PDx	0.0 ± 0.0^a^	2.1 ± 2.1^a^	17.4 ± 3.1^b^
	14-HDoHE	1,754.9 ± 383.6	1,743.4 ± 323.6	2,961.9 ± 354.3
	17-HDoHE	2486.1 ± 646.4	2178.8 ± 429.8	3218.1 ± 395.3

*The oxidized metabolites of fatty acids were quantified in heart by LC-QQQ as mentioned in methods*.

a, b, c and ab *indicate significant differences between the three groups when p < 0.05; ab means no difference with a and no difference with b*.

*Colored values correspond to the type of omelets: black for control, red for UN-DHA-O and blue for EN-DHA-O*.

At last, in brain, no effect of the DHA oil supplementation was observed on measured oxidized metabolites (Table 8). But more interestingly, three oxylipins were significantly increased when the DHA oil was encapsulated. Therefore, 14-HDoHE derived from DHA was almost doubled with EN-DHA-O as compared with the control and the UN-DHA-O groups, while 8-HETE and 5,6-EET, positively correlated in brain (R^2^ = 0.5845, *p* < 0.001, not shown), were more reasonably raised with EN-DHA-O. To conclude, the DHA supplementation globally modulated the oxylipin pattern of tissues, but the modulation was particularly prominent when the DHA oil was encapsulated.

**Table 8:** The oxidized derivatives of fatty acids from brain.

**pg/mg protein**		**Control**	**UN-DHA-O**	**EN-DHA-O**
LA	13-HODE	103.2 ± 14.2	108.6 ± 12.0	154.0 ± 19.6
DGLA	6kPGF_1α_	272.3 ± 39.7	324.4 ± 57.6	428.7 ± 64.2
ARA	PGF_2α_	3,641.1 ± 367.7	4,062.1 ± 266.9	4,184.5 ± 187.4
	PGE_2_	401.0 ± 39.8	499.0 ± 40.3	540.3 ± 71.4
	PGD_2_	3,040.4 ± 249.4	3,679.7 ± 307.1	3,644.2 ± 254.5
	TXB_2_	577.0 ± 53.4	637.4 ± 53.3	715.8 ± 109.6
	15-HETE	716.0 ± 69.6	782.2 ± 65.2	861.5 ± 52.1
	8-HETE	147.3 ± 15.0^a^	151.8 ± 8.3^a^	218.9 ± 25.5^b^
	12-HETE	3557.6 ± 438.4	4908.4 ± 679.7	5055.1 ± 472.0
	5-HETE	840.3 ± 85.9	834.8 ± 72.2	1046.5 ± 206.8
	14,15-EET	223.4 ± 20.5	217.3 ± 22.3	272.9 ± 37.8
	5-oxo-ETE	588.5 ± 75.7	569.6 ± 66.6	705.5 ± 55.3
	11,12-EET	279.2 ± 36.6	267.3 ± 26.1	297.5 ± 47.1
	8,9-EET	380.9 ± 44.4	370.4 ± 33.9	554.7 ± 95.2
	5,6-EET	566.1 ± 90.9^ab^	446.0 ± 54.2^b^	730.1 ± 73.0^a^
DHA	14-HDoHE	382.6 ± 47.1^a^	385.5 ± 63.2^a^	597.1 ± 61.1^b^
	17-HDoHE	375.8 ± 49.1	581.6 ± 113.7	645.8 ± 55.4

*The oxidized metabolites of fatty acids were quantified in brain by LC-QQQ as mentioned in methods*.

a, b, c and ab *indicate significant differences between the three groups when p < 0.05; ab means no difference with a and no difference with b*.

*Colored values correspond to the type of omelets: black for control, red for UN-DHA-O and blue for EN-DHA-O*.

## Discussion

In this study, DHA esterified to TAG was encapsulated with natural whey proteins in producing Pickering emulsion. Egg was chosen as a food matrix to favor the bioavailability of DHA (36), but we used cooked egg as omelet to determine the tissue accretion of encapsulated DHA in using rats as experimental model. Overall, the DHA supplementation increased the DHA concentration in tissues except brain and eyes. EPA was increased as well in plasma, RBC, and liver. This FA was possibly synthesized from n-3 precursors such as ALA present in T-diet. Nevertheless, this raise was inherent to the DHA supplementation in omelets, considering that EPA was minorly present in DHA oil and was potentially generated by retro-conversion of DHA or of DPA n-3. DPA n-3 was also minorly present in DHA oil, to the same extent than EPA. However, its metabolism differed since DPA n-3 was weakly accumulated in tissues. As EPA was directly provided by egg food and easily synthesized from ALA, retro-conversion may not be favored in our conditions of work, although the importance of retro-conversion to produce EPA is still under discussion (41–43). Now, considering more specifically DHA, accretion was measured whereas rats were fed from weaning during 4 weeks of growth with an optimized diet. In these conditions, the DHA supplementation did not improve significantly DHA accretion in brain, as the DHA concentration was already high in the tissue, reaching more than 20% of total FA regardless of the diet. Our result contrasts with other studies where DHA proportion in brain was significantly increased by around 20% when rats fed a DHA enriched-diet as compared with the control (19, 44–46). First, it has to be mentioned that important differences between studies were also highlighted by other authors concerning mouse models, where comparisons were difficult considering strains, brain regions, ages, and diet conditions (47). Then, it must be noted that this effect was observed in the literature while the DHA proportion in brain was usually close to 10–15% of the total FA, which greatly differs from our DHA proportion reaching 22.8% in the control group. Additionally, many of the studies on DHA were designed with a low n-3 PUFA diet, favoring DHA half-life in brain and synthesis rate from the liver. In our study, the experimental design favored a higher concentration of DHA in frontal cortex, which was not significantly improved by the dietary DHA supplementation. This effect may result from ALA supplied from the T-diet in complement to the low level of PL-DHA from eggs. It was indeed shown that DHA synthesis from ALA may be sufficient to maintain brain DHA concentrations in the absence of dietary DHA consumption (48). Here, a high level of DHA in brain was possibly conditioned by an important *de novo* synthesis from dietary ALA and favored by the daily fasting as well. Short-term fasting was shown to enhance DHA concentrations in serum without dietary supplementation with DHA (49). The authors suggested that an increase in the expression of FA transporters specific for DHA likely mediated an enrichment of PUFA in hepatic TAG. This enrichment may foster secretion of DHA from the liver as 2-DHA-lysoPL for recirculation, subsequently promoting brain DHA accretion. Hepatic lipase was actually shown to play a major role in generating 1-lyso-2-DHA-*sn*-glycerophosphocholine, one of the main forms of DHA transported through the blood brain barrier for brain DHA enrichment (50). However, in our study, encapsulation of dietary DHA was shown to significantly improve neither bioavailability nor brain accretion of DHA. This result contrasts with other works based on whey protein or lipid emulsions. When rats were treated by gavage with encapsulated linseed oil, DHA was enhanced in cardiac sarcoplasmic reticulum by 28 and 94% with protein and phospholipid-based emulsions, respectively (51). When DHA was supplied as fish oil, the DHA was more accumulated in tissues with encapsulated oil than with native oil (52). Nevertheless, the effect was high with lipid emulsion and much less important with whey protein emulsion. More specifically in brain, the DHA accretion was upgraded from 11.4% with native fish oil to 12.6% with whey protein encapsulation. If the nature of encapsulation plays a major role in the tissue delivery of DHA, methods of administration may impact the bioavailability of DHA as well. In many studies, animals were fed encapsulated oil by gavage. In this study, we integrated functional food with a cook processing. The daily preparation was constraining but, in that way, omelets were baked with natural compounds and no additives.

In view of the results on FA profiles, we further analyzed DMA derived from plasmalogens. These etherlipids, and more precisely those derived from phosphatidylethanolamine, are known as carriers of long-chain PUFA in brain. Zhao et al. showed in mouse cortex that plasmenylethanolamine was composed of 30% DHA, 25% DPAn-3, and 10% EPA (53). In brain, we found the major alkenyl chains described in plasmalogen species, as the authors highlighted that plasmenylethanolamine mainly contained 18:0 and 16:0 at the *sn*-1 position when DHA was esterified at the *sn*-2 position. But overall, our diets did not change the DMA profile in brain, heart, or RBC, although the DHA proportion was increased in heart and RBC with the DHA supplementation. A previous work showed that the plasmalogen content in brain may be increased by dietary DHA through a potent stimulation of dihydroxyacetone phosphate acyl transferase activity (54). This effect was however presumed as relevant at the beginning of the life but not during aging. In our study, DHA supplementation consistently affected the DMA structures and concentrations in eyes, but surprisingly in reducing the DMA content. We also found equivalent proportions between saturated DMA (around 35% for both 16:0-DMA and 18:0-DMA) and between monounsaturated DMA (around 15% for both 18:1n-9-DMA and 18:1n-7-DMA), on the contrary to other studies performed on mice (55), where the diet enriched in fat modulated as well the DMA profile. DHA supplementation may shift the balance between plasmalogens, since few phospholipid species coexist in retina and other structures in eyes (56). Some authors showed in mouse cerebral cortex that DHA-enriched diet reduced the n-6 PUFA content in plasmenylethanolamine, the concentration of which tended to decrease in the tissue, whereas a substantial raise in peroxidation products was observed as well (57). Considering all the data, high concentrations of DHA in eye may explain a lower content of plasmalogens, which are considered as scavenger agents to protect against oxidation (58). Nevertheless, the discrepancy between control and DHA-enriched diets remains difficult to explain. The regulation of dietary FA on enzymes involved in plasmalogen metabolism has to be further investigated.

Finally, many studies showed that DHA enriched-diets modulate the production of mediators derived from FA such as oxylipins. Oxidized metabolites are miscellaneous and mainly generated by cyclooxygenase, lipoxygenase, and cytochrome P450 (23). In this study, we first determined the oxylipin pattern of DHA oil. We found eight compounds primarily derived from LA with 13- and 9-HODE, and from DHA with 17- and 14-HDoHE. When quantification was performed on omelets, half of the oxylipins were lost. Only 13-HODE, 9-HODE, and 5-HETE were still quantified. DHA supplementation was not discriminant as the oxylipin profile was similar between the three omelets. However, in DHA-enriched omelets, all oxylipins were expected in spite of the small amount of DHA oil added in egg preparation. Thus, this result suggests that food processing destroyed most of the oxidized derivatives of PUFA supplied by DHA oil in DHA-enriched omelets, while promoting non-enzymatic oxidation by generating specific hydroxylated FA such as HODE and HETE from egg FA. In such conditions, all groups of animals fed a similar amount of oxylipins derived from omelets. Quantification of oxylipins was performed on one set of food, but we may consider a consumption of 8 to 15 ng oxylipins per day per rat. On this basis, we further evaluated the impact of DHA encapsulation. Our data showed that DHA supplementation modified the oxylipin patterns in plasma and heart, and to a lesser extent in brain. The dietary effect on n-6 metabolites dominated over the effect on docosanoids generated from DHA. But most importantly, we demonstrated a very high influence of DHA oil encapsulation on the oxylipin concentrations in tissues, even in brain. Precisely, 14-HDoHE was drastically reduced in plasma and raised in brain with DHA encapsulation. 14-HDoHE is subsequently generated by 12-lipoxygenase and glutathione peroxidase (59). Other studies showed an increase in 14-HDoHE consecutive to a n-3 enriched-diet in brain (20, 60) or in plasma (61, 62). Blood concentration was enhanced in pathological conditions such as morbid obesity (63) or metabolic syndrome (61), whereas the local level of 14-HDoHE was usually reduced in inflammatory conditions such as in arthritis (64), periodontitis (65), or pulmonary inflammation (66). 14-HDoHE was shown to be involved in the resolution of inflammation, to participate in the immune response or the platelet activation in reducing thrombus formation (67, 68). If the mechanism of action of 14-HDoHE still remains unclear, a cerebral increase in 14-HDoHE may be a good marker to prevent potent neuroinflammation. In heart, the level of 14-HDoHE tended to increase but more importantly, the accumulation of PDx was favored with DHA oil encapsulation. This poxytrin is generated by the action of 15-lipoxygenase on DHA and displays a pleiotropic function in inhibiting inflammation, cyclooxygenases 1 and 2, production of reactive oxygen species, and RNA virus replication (69). PDx also improved the liver steatosis status (70) and lowered hepatic gluconeogenesis in an obese mouse model (71), making it a potential therapeutic agent for lipid-induced and obesity-linked insulin resistance and type 2 diabetes (72). More specifically, PDx may be a resolution mediator of kidney fibrosis and cardiac failure (73). Only healthy functions have been attributed to PDx, which makes the DHA supplementation a valuable approach to improve health tissue before potential injury. DHA oil used in our study was prepared enzymatically by esterification of glycerol with enriched DHA oil extracted from fish. The DHA content was high but other PUFA such as EPA was lowly detected. Although minorly present, EPA from the DHA-enriched diets displayed a remarkable impact on cardiac tissues as we found 18-HEPE with DHA supplementation but only when DHA oil was encapsulated. 18-HEPE is generated from EPA through catalysis of COX-2/aspirin or cytochrome P450 as a precursor of resolvin E1. It accumulated as well in brain (60) or plasma (61, 62) after a diet enriched in n-3 PUFA. Its blood concentration decreased with the obesity-linked inflammation status (61, 63), whereas it raised in inflammatory arthritis (64). 18-HEPE was also particularly concentrated in plasma of fat-1 mice compared with wild-type mice, and it was shown to protect against pulmonary metastasis of melanoma (74). More specifically, 18-HEPE was highly correlated with the coronary plaque regression on patients with coronary artery disease supplemented with high dose of n-3 PUFA for 30 months (75). Collectively in heart, the increase in PDx and 18-HEPE, together with the reduction of n-6 eicosanoids triggered by both DHA supply and oil encapsulation, emphasized the upgraded status on cardiac tissues on a short-term supplementation. Encapsulation of DHA oil with whey protein as Pickering emulsion showed in our study that the delivery system was efficient to modulate derivatives of PUFA in tissues, as compared to non-encapsulated DHA oil. This effect was presumed to be mediated by a different rate of absorption of DHA oil. Indeed, when the same omelets were digested by using an *in vitro* model of static digestion for adults, lipolysis of DHA-TAG was enhanced when DHA oil was encapsulated (unpublished result). Emulsions presenting the lowest droplet size prior to digestion exhibit a better rate of lipolysis compared to coarser emulsion (76). The same observation was assigned to nanoemulsion as compared with microemulsion where the release of oil took place either by interfacial transfer or degradation of the vehicle driving the lipids out (77). The breakdown of encapsulated oil depends as well on differences in emulsion structure and surface area available for catalytic activity. Many encapsulant materials are available (78) but the best delivery system of marine oil has to be developed and experienced. The subsequent lipolysis rate and thus absorption into the intestine greatly influenced the bioavailability of DHA as demonstrated with volunteers submitted to fish oil supplementation (79, 80).

Reducing lipid droplet size affects satiety as well by increasing FA sensing in small intestine or by enhancing peptide secretion like cholecystokinin or peptide YY during digestion (81). In our work, encapsulation of DHA oil heightened the growth of animals coupled with a change in eating behavior. We observed that the rats were particularly enthusiastic to eat DHA-enriched omelets and especially in the EN-DHA-O group. Yet, a study on volunteers showed that palatability was negatively impacted by the highest concentrations of DHA when fish oil was added to ground beef. Moreover, panelists were distinctly sensitive depending on the FA as EPA had a greater impact on off-flavor perception than DHA (82). Nonetheless, contrary to the fishy flavors triggered by bulk fish oil, more sophisticated preparation of oil such as encapsulation may improve palatability of food. Encapsulating DHA oil possibly mitigated the unpleasant taste leading to a better acceptance of DHA-enriched omelet. Now, the origin of oil may influence food intake as well. In considering a longer-term supplementation, the eating behavior was shown to be influenced by the effect of DHA on the reward system regulating appetite (83). Chalon et al. demonstrated that the mesolimbic dopaminergic pathway was overactivated in rats deficient in n-3 PUFA (84). Lacking n-3 PUFA increased reward sensitivity as well in patients with intrauterine growth restriction. This pathology affects brain responses to palatable food creating an impulsive eating behavior, reversible with n-3 PUFA supplementation (85). The alimentary impact was partly explained by the leptin pathway involved in the anorexigenic system. Indeed, DHA-TAG enhanced leptin secretion in obese mice, consequently reducing hyperphagia in regulating appetite (86). Conversely, one study counteracted this data on patients with haemodialysis suffering from anorexia (87). Implying of DHA was suggested to be attributed to its derivatives such as the endocannabinoid docosahexaenoyl ethanolamide (DHEA). Positively correlated to appetite, DHEA was suspected to act as an activator of the orexigenic pathway, possibly by antagonizing the putative anorectic effects of linoleoyl ethanolamide. If data are controversial in presenting DHA or derivatives either as an enhancer or as an inhibitor of appetite, other effects on food reward circuit are still poorly described. Aside from the neuronal implication, DHEA was otherwise shown to improve glucose intake of myoblasts *in vitro* through the activation of the endocannabinoid system (88). A similar result was observed on sows-fed DHA-enriched fish oil during gestation. Intestinal glucose uptake was therefore increased in offspring, in association with the n-3 PUFA enrichment of jejunum tissues (89). This effect was partly mediated by the raise in brush border membrane glucose transporters. Transposed to our work, the effect of DHA encapsulation on animal growth was possibly facilitated by the modulation of intestinal transport functions as well. All together, these data suggest that encapsulation of DHA oil may upgrade the food palatability of omelets, improve appetite for the T-diet, and favor growth in promoting energy metabolism. For as much, it remains difficult to interpret the effect of encapsulation observed on derivatives of PUFA, more than on PUFAs themselves. The molecular mechanism requires further research for understanding.

To conclude, we used a rat model to assess the efficacy of a food-graded delivery system by encapsulating DHA oil into whey protein. In our experimental conditions, the brain displayed a great proportion of 22.8% DHA, which was not enhanced by dietary supplementation of DHA oil, as compared to other tissues. Overall, the encapsulation of DHA oil did not modulate the FA profile as compared to the literature, but remarkably modified the oxylipin pattern in plasma, heart, and even brain. Specific oxidized metabolites derived from DHA were upgraded while those from n-6 PUFA were essentially mitigated. This effect was independent of oxylipins present in DHA oil as food processing induced the loss of the specific oxidized metabolites from DHA and EPA. Finally, this work showed that encapsulation of DHA oil remains a key factor for DHA metabolism in generating precursors of protectins and maresins, thus improving the global health status.

## Data Availability Statement

The original contributions presented in the study are included in the article/Supplementary Material, further inquiries can be directed to the corresponding author/s.

## Ethics Statement

The animal study was reviewed and approved by French Ministry of Higher Education and Research.

## Author Contributions

DD and FP conceived the study. FP designed the experimental work. JW performed the experiments with the help of JO, YL, and FB. JW and FP analyzed the data and wrote the manuscript. DD reviewed the manuscript. All authors contributed to the article and approved the submitted version.

## Conflict of Interest

The authors declare that the research was conducted in the absence of any commercial or financial relationships that could be construed as a potential conflict of interest.

## Publisher's Note

All claims expressed in this article are solely those of the authors and do not necessarily represent those of their affiliated organizations, or those of the publisher, the editors and the reviewers. Any product that may be evaluated in this article, or claim that may be made by its manufacturer, is not guaranteed or endorsed by the publisher.

## Abbreviations

ALA: α-Linolenic acid (18:3n-3)
ARA: Arachidonic acid (20:4n-6)
Control: omelet without DHA supplementation
DGLA: Dihomo-α-linolenic acid (20:3n-6)
DHA: Docosahexaenoic acid (22:6n-3)
DHEA: Docosahexaenoyl ethanolamide
DMA: Dimethylacetal
DPA: Docosapentaenoic acid (22:5n-3)
5,6-EET: (±)5(6)-Epoxy-8Z,11Z,14Z-eicosatrienoic acid
8,9-EET: (±)8(9)-Epoxy-5Z,11Z,14Z-eicosatrienoic acid
11,12-EET: (±)11(12)-Epoxy-5Z,8Z,14Z-eicosatrienoic acid
14,15-EET: (±)14(15)-Epoxy-5Z,8Z,11Z-eicosatrienoic acid
EN-DHA-O: Encapsulated DHA oil in omelet
EPA: Eicosapentaenoic acid (20:5n-3)
FA: Fatty acid
FAME: Fatty acid methyl ester
H-diet: Habituation diet
14-HDoHE: (±)14-Hydroxy-4Z,7Z,10Z,12E,16Z,19Z-docosahexaenoic acid
17-HDoHE: (±)17-Hydroxy-4Z,7Z,10Z,13Z,15E,19Z-docosahexaenoic acid
18-HEPE: (±)18-Hydroxy-5Z,8Z,11Z,14Z,16E-eicosapentaenoic acid
5-HETE: 5(S)-Hydroxy-5Z,8Z,11Z,13E-eicosatetraenoic acid
8-HETE: (±)8-Hydroxy-5Z,9E,11Z,14Z-eicosatetraenoic acid
12-HETE: 12(S)-Hydroxy-5Z,8Z,10E,14Z-eicosatetraenoic acid
15-HETE: 15(S)-Hydroxy-5Z,8Z,11Z,13E-eicosatetraenoic acid
9-HODE: 9-Hydroxy-10E,12Z-octadecadienoic acid
13-HODE: 13-Hydroxy-9Z,11E-octadecadienoic acid
LA: Linoleic acid (18:2n-6)
5-oxo-ETE: 5-oxo-6E,8Z,11Z,14Z-eicosatetraenoic acid
PGD_2_: Prostaglandin D_2_
PDx: Protectin Dx
PGE_2_: Prostaglandin E_2_
6kPGF_1_α: 6-keto prostaglandin F_1_α
PGF_2_α: Prostaglandin F_2_α
15d-PGJ_2_: 15-deoxy-delta-12,14-prostaglandin J_2_
PL: Phospholipid
PUFA: Polyunsaturated fatty acids
RBC: Red blood cells
TAG: Triacylglycerol
T-diet: Treatment diet
TXB_2_: Thromboxane B_2_
UN-DHA-O: Unencapsulated DHA oil in omelet.
