# Supplementary material for: Encapsulation of Docosahexaenoic Acid Oil Substantially Improves the Oxylipin Profile of Rat Tissues
Source: Front Nutr. 2022 Jan 13;8:812119. doi: 10.3389/fnut.2021.812119 (PMC8805515; doi:10.3389/fnut.2021.812119)
Supplement: Supplementary file 1 [file Data_Sheet_1.PDF]

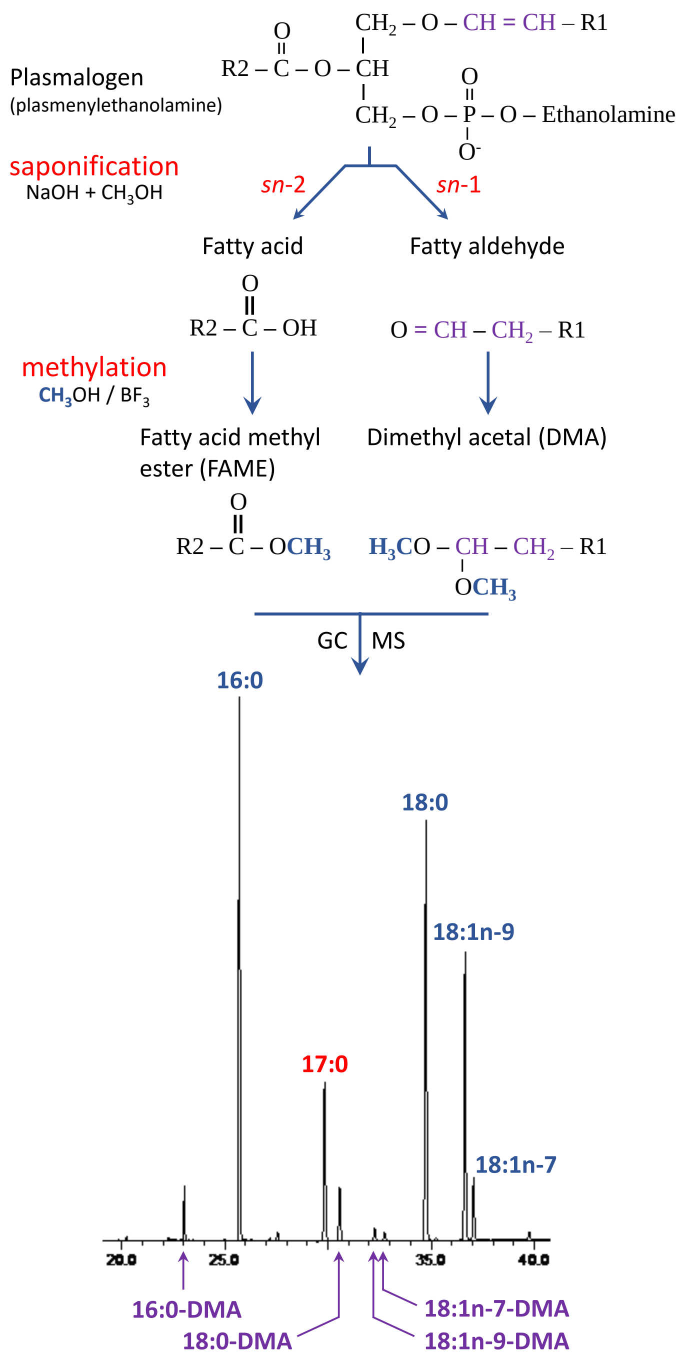


**Supplemental Figure 1. Dimethylacetal formation from plasmalogens after saponification and methylation.**

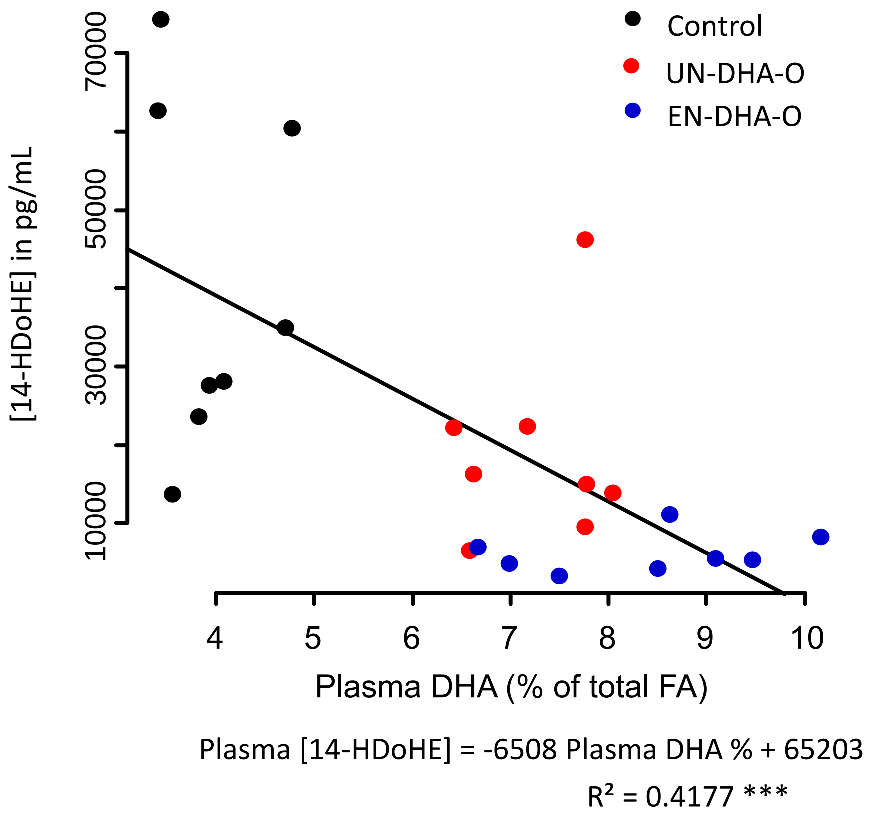


**Supplemental Figure 2. Correlation between 14-HDoHE concentrations in plasma and DHA proportions in plasma.**

Pearson correlation was determined from plasma measurements of DHA and 14-HDoHE from all the data. Omelets were identified by colored symbols.

**Supplemental Table 1. The fatty acid profile of plasma and RBC.**

Lipids of plasma and red blood cells were extracted by the Folch's method. The fatty acid profile was determined by GC-MS and expressed in mass %.

| % | Plasma | | | RBC | | |
|---|---|---|---|---|---|---|
| | Control | UN-DHA-O | EN-DHA-O | Control | UN-DHA-O | EN-DHA-O |
| 12:0 | 0.1 ± 0.0 | 0.0 ± 0.0 | 0.0 ± 0.0 | 0.0 ± 0.0 | 0.0 ± 0.0 | 0.0 ± 0.0 |
| 14:0 | 0.5 ± 0.0 | 0.5 ± 0.0 | 0.5 ± 0.0 | 0.2 ± 0.0 | 0.2 ± 0.0 | 0.2 ± 0.0 |
| 16:0 | 21.0 ± 0.3 $^{a}$ | 22.0 ± 0.3 $^{ab}$ | 22.5 ± 0.2 $^{b}$ | 27.5 ± 0.5 | 28.8 ± 0.3 | 28.1 ± 0.3 |
| 18:0 | 7.7 ± 0.1 | 7.5 ± 0.4 | 7.4 ± 0.1 | 11.0 ± 0.2 | 11.2 ± 0.1 | 11.6 ± 0.3 |
| 20:0 | 0.0 ± 0.0 | 0.0 ± 0.0 | 0.0 ± 0.0 | 0.0 ± 0.0 | 0.0 ± 0.0 | 0.0 ± 0.0 |
| 22:0 | 0.0 ± 0.0 | 0.0 ± 0.0 | 0.0 ± 0.0 | 0.0 ± 0.0 | 0.0 ± 0.0 | 0.0 ± 0.0 |
| 24:0 | 0.0 ± 0.0 | 0.0 ± 0.0 | 0.0 ± 0.0 | 0.0 ± 0.0 | 0.0 ± 0.0 | 0.0 ± 0.0 |
| **saturated** | **29.3 ± 0.3** $^{a}$ | **30.0 ± 0.1** $^{ab}$ | **30.4 ± 0.2** $^{b}$ | **38.7 ± 0.6** $^{a}$ | **40.2 ± 0.2** $^{b}$ | **39.9 ± 0.2** $^{ab}$ |
| 16:1n-7 | 3.2 ± 0.1 | 2.9 ± 0.2 | 2.9 ± 0.1 | 0.8 ± 0.1 $^{a}$ | 0.5 ± 0.0 $^{b}$ | 0.5 ± 0.0 $^{b}$ |
| 18:1n-7 | 2.5 ± 0.1 $^{a}$ | 2.0 ± 0.1 $^{b}$ | 2.0 ± 0.1 $^{b}$ | 3.2 ± 0.0 $^{a}$ | 3.0 ± 0.1 $^{b}$ | 3.0 ± 0.1 $^{b}$ |
| 20:1n-7 | 0.2 ± 0.0 | 0.2 ± 0.0 | 0.1 ± 0.0 | 0.1 ± 0.0 | 0.0 ± 0.0 | 0.0 ± 0.0 |
| **n-7** | **5.9 ± 0.1** $^{a}$ | **5.1 ± 0.2** $^{b}$ | **5.0 ± 0.1** $^{b}$ | **4.1 ± 0.1** $^{a}$ | **3.5 ± 0.1** $^{b}$ | **3.5 ± 0.1** $^{b}$ |
| 16:1n-9 | 0.2 ± 0.0 | 0.2 ± 0.0 | 0.2 ± 0.0 | 0.1 ± 0.0 | 0.1 ± 0.0 | 0.1 ± 0.0 |
| 18:1n-9 | 18.6 ± 0.5 | 19.0 ± 0.8 | 19.6 ± 0.4 | 9.4 ± 0.3 | 9.3 ± 0.2 | 8.8 ± 0.2 |
| 20:1n-9 | 0.1 ± 0.0 | 0.0 ± 0.0 | 0.0 ± 0.0 | 0.1 ± 0.0 | 0.1 ± 0.0 | 0.1 ± 0.0 |
| 20:2n-9 | 0.0 ± 0.0 | 0.0 ± 0.0 | 0.0 ± 0.0 | 0.0 ± 0.0 | 0.0 ± 0.0 | 0.0 ± 0.0 |
| 20:3n-9 | 0.2 ± 0.0 $^{a}$ | 0.1 ± 0.0 $^{b}$ | 0.1 ± 0.0 $^{b}$ | 0.1 ± 0.0 | 0.1 ± 0.0 | 0.1 ± 0.0 |
| 22:1n-9 | 0.0 ± 0.0 | 0.0 ± 0.0 | 0.0 ± 0.0 | 0.0 ± 0.0 | 0.0 ± 0.0 | 0.0 ± 0.0 |
| 24:1n-9 | 0.0 ± 0.0 | 0.0 ± 0.0 | 0.0 ± 0.0 | 0.0 ± 0.0 | 0.0 ± 0.0 | 0.0 ± 0.0 |
| **n-9** | **19.1 ± 0.5** | **19.3 ± 0.8** | **19.9 ± 0.4** | **9.7 ± 0.3** | **9.6 ± 0.2** | **9.1 ± 0.2** |
| 18:2n-6 | 11.1 ± 0.2 | 11.7 ± 0.1 | 11.1 ± 0.3 | 7.0 ± 0.2 | 7.4 ± 0.1 | 7.0 ± 0.2 |
| 18:3n-6 | 0.1 ± 0.0 | 0.1 ± 0.0 | 0.0 ± 0.0 | 0.0 ± 0.0 | 0.0 ± 0.0 | 0.0 ± 0.0 |
| 20:2n-6 | 0.0 ± 0.0 | 0.0 ± 0.0 | 0.0 ± 0.0 | 0.1 ± 0.0 | 0.2 ± 0.0 | 0.2 ± 0.0 |
| 20:3n-6 | 0.3 ± 0.0 | 0.4 ± 0.0 | 0.4 ± 0.0 | 0.6 ± 0.0 | 0.6 ± 0.0 | 0.6 ± 0.0 |
| 20:4n-6 | 28.3 ± 0.7 $^{a}$ | 23.5 ± 1.0 $^{b}$ | 21.9 ± 0.3 $^{b}$ | 31.5 ± 0.2 $^{a}$ | 28.3 ± 0.2 $^{b}$ | 28.8 ± 0.2 $^{b}$ |
| 22:4n-6 | 0.2 ± 0.0 $^{a}$ | 0.0 ± 0.0 $^{b}$ | 0.1 ± 0.0 $^{b}$ | 1.3 ± 0.0 $^{a}$ | 0.8 ± 0.0 $^{b}$ | 0.8 ± 0.0 $^{b}$ |
| 22:5n-6 | 0.2 ± 0.0 $^{a}$ | 0.1 ± 0.0 $^{b}$ | 0.1 ± 0.0 $^{b}$ | 0.5 ± 0.0 $^{a}$ | 0.3 ± 0.0 $^{b}$ | 0.3 ± 0.0 $^{b}$ |
| **n-6** | **40.2 ± 0.5** $^{a}$ | **35.8 ± 1.0** $^{b}$ | **33.6 ± 0.3** $^{b}$ | **41.0 ± 0.4** $^{a}$ | **37.6 ± 0.2** $^{b}$ | **37.7 ± 0.2** $^{b}$ |
| 18:3n-3 | 0.6 ± 0.0 | 0.6 ± 0.0 | 0.6 ± 0.0 | 0.1 ± 0.0 | 0.0 ± 0.0 | 0.0 ± 0.0 |
| 20:5n-3 | 0.5 ± 0.0 $^{a}$ | 1.4 ± 0.1 $^{b}$ | 1.4 ± 0.1 $^{b}$ | 0.3 ± 0.0 $^{a}$ | 0.9 ± 0.0 $^{b}$ | 0.9 ± 0.0 $^{b}$ |
| 22:5n-3 | 0.4 ± 0.0 $^{a}$ | 0.6 ± 0.1 $^{b}$ | 0.8 ± 0.1 $^{b}$ | 1.7 ± 0.1 $^{a}$ | 1.9 ± 0.1 $^{ab}$ | 2.0 ± 0.0 $^{b}$ |
| 22:6n-3 | 3.9 ± 0.2 $^{a}$ | 7.2 ± 0.2 $^{b}$ | 8.3 ± 0.4 $^{b}$ | 4.2 ± 0.1 $^{a}$ | 6.1 ± 0.1 $^{b}$ | 6.7 ± 0.1 $^{c}$ |
| **n-3** | **5.4 ± 0.2** $^{a}$ | **9.8 ± 0.3** $^{b}$ | **11.1 ± 0.5** $^{b}$ | **6.3 ± 0.1** $^{a}$ | **8.9 ± 0.1** $^{b}$ | **9.6 ± 0.1** $^{c}$ |
| **others** | **0.1 ± 0.0** | **0.0 ± 0.0** | **0.0 ± 0.0** | **0.2 ± 0.0** | **0.2 ± 0.0** | **0.2 ± 0.0** |
| μg/mg | 1.9 ± 0.1 | 1.9 ± 0.1 | 2.0 ± 0.1 | 2.2 ± 0.0 | 2.1 ± 0.0 | 2.2 ± 0.0 |

**Supplemental Table 2. The fatty acid profile of liver and heart.**

Lipids were extracted by the Folch’s method. The fatty acid profile was determined by GC-MS and expressed in mass %.

| % | Liver | | | Heart | | |
|---|---|---|---|---|---|---|
| | Control | UN-DHA-O | EN-DHA-O | Control | UN-DHA-O | EN-DHA-O |
| 12:0 | 0.0 ± 0.0 | 0.0 ± 0.0 | 0.0 ± 0.0 | 0.0 ± 0.0 | 0.0 ± 0.0 | 0.0 ± 0.0 |
| 14:0 | 0.5 ± 0.0 [a] | 0.4 ± 0.0 [b] | 0.5 ± 0.0 [ab] | 0.1 ± 0.0 | 0.1 ± 0.0 | 0.1 ± 0.0 |
| 16:0 | 22.1 ± 0.4 [a] | 22.3 ± 0.5 [a] | 24.2 ± 0.4 [b] | 11.3 ± 0.1 | 11.9 ± 0.2 | 11.9 ± 0.2 |
| 18:0 | 10.4 ± 0.4 | 11.3 ± 0.7 | 10.7 ± 0.8 | 20.6 ± 0.4 | 20.1 ± 0.3 | 20.4 ± 0.5 |
| 20:0 | 0.0 ± 0.0 | 0.0 ± 0.0 | 0.0 ± 0.0 | 0.0 ± 0.0 | 0.0 ± 0.0 | 0.0 ± 0.0 |
| 22:0 | 0.0 ± 0.0 | 0.0 ± 0.0 | 0.0 ± 0.0 | 0.0 ± 0.0 | 0.0 ± 0.0 | 0.0 ± 0.0 |
| 24:0 | 0.0 ± 0.0 | 0.0 ± 0.0 | 0.0 ± 0.0 | 0.0 ± 0.0 | 0.0 ± 0.0 | 0.0 ± 0.0 |
| **saturated** | **33.0 ± 0.4** | **34.0 ± 0.6** | **35.4 ± 0.8** | **32.0 ± 0.4** | **32.1 ± 0.4** | **32.4 ± 0.5** |
| 16:1n-7 | 3.7 ± 0.2 [a] | 2.7 ± 0.2 [b] | 3.4 ± 0.3 [a] | 0.4 ± 0.0 | 0.4 ± 0.0 | 0.3 ± 0.0 |
| 18:1n-7 | 3.6 ± 0.1 [a] | 2.9 ± 0.1 [b] | 3.0 ± 0.2 [b] | 3.7 ± 0.0 | 3.5 ± 0.1 | 3.7 ± 0.1 |
| 20:1n-7 | 0.1 ± 0.0 | 0.1 ± 0.0 | 0.0 ± 0.0 | 0.0 ± 0.0 | 0.0 ± 0.0 | 0.0 ± 0.0 |
| **n-7** | **7.4 ± 0.2 [a]** | **5.7 ± 0.3 [b]** | **6.4 ± 0.4 [ab]** | **4.1 ± 0.1** | **3.9 ± 0.1** | **4.0 ± 0.1** |
| 16:1n-9 | 0.3 ± 0.0 | 0.3 ± 0.0 | 0.3 ± 0.0 | 0.0 ± 0.0 | 0.0 ± 0.0 | 0.0 ± 0.0 |
| 18:1n-9 | 22.6 ± 0.9 | 20.6 ± 1.0 | 23.0 ± 1.3 | 5.6 ± 0.1 | 6.1 ± 0.3 | 5.1 ± 0.3 |
| 20:1n-9 | 0.1 ± 0.0 | 0.1 ± 0.0 | 0.1 ± 0.0 | 0.0 ± 0.0 | 0.0 ± 0.0 | 0.0 ± 0.0 |
| 20:2n-9 | 0.0 ± 0.0 | 0.0 ± 0.0 | 0.0 ± 0.0 | 0.0 ± 0.0 | 0.0 ± 0.0 | 0.0 ± 0.0 |
| 20:3n-9 | 0.2 ± 0.0 | 0.1 ± 0.0 | 0.1 ± 0.0 | 0.0 ± 0.0 | 0.0 ± 0.0 | 0.0 ± 0.0 |
| 22:1n-9 | 0.0 ± 0.0 | 0.0 ± 0.0 | 0.0 ± 0.0 | 0.0 ± 0.0 | 0.0 ± 0.0 | 0.0 ± 0.0 |
| 24:1n-9 | 0.0 ± 0.0 | 0.0 ± 0.0 | 0.0 ± 0.0 | 0.0 ± 0.0 | 0.0 ± 0.0 | 0.0 ± 0.0 |
| **n-9** | **23.2 ± 0.9** | **21.1 ± 1.1** | **23.5 ± 1.3** | **5.6 ± 0.1** | **6.1 ± 0.3** | **5.1 ± 0.3** |
| 18:2n-6 | 9.3 ± 0.3 | 10.0 ± 0.2 | 8.6 ± 0.4 | 15.1 ± 0.4 [a] | 13.1 ± 0.4 [b] | 12.4 ± 0.5 [b] |
| 18:3n-6 | 0.1 ± 0.0 | 0.1 ± 0.0 | 0.1 ± 0.0 | 0.0 ± 0.0 | 0.0 ± 0.0 | 0.0 ± 0.0 |
| 20:2n-6 | 0.1 ± 0.0 | 0.0 ± 0.0 | 0.0 ± 0.0 | 0.0 ± 0.0 | 0.0 ± 0.0 | 0.0 ± 0.0 |
| 20:3n-6 | 0.4 ± 0.0 | 0.5 ± 0.0 | 0.4 ± 0.0 | 0.2 ± 0.0 | 0.3 ± 0.0 | 0.3 ± 0.0 |
| 20:4n-6 | 18.9 ± 0.8 [a] | 17.3 ± 0.7 [ab] | 15.5 ± 0.9 [b] | 27.2 ± 0.3 [a] | 22.7 ± 0.6 [b] | 22.6 ± 0.5 [b] |
| 22:4n-6 | 0.2 ± 0.0 [a] | 0.1 ± 0.0 [b] | 0.1 ± 0.0 [b] | 0.7 ± 0.0 [a] | 0.3 ± 0.0 [b] | 0.4 ± 0.0 [b] |
| 22:5n-6 | 0.2 ± 0.0 [a] | 0.1 ± 0.0 [b] | 0.1 ± 0.0 [b] | 0.9 ± 0.1 [a] | 0.4 ± 0.0 [b] | 0.5 ± 0.0 [b] |
| **n-6** | **29.2 ± 0.8 [a]** | **28.1 ± 0.8 [a]** | **24.8 ± 0.9 [b]** | **44.1 ± 0.4 [a]** | **36.8 ± 0.3 [b]** | **36.0 ± 0.5 [b]** |
| 18:3n-3 | 0.5 ± 0.0 | 0.6 ± 0.0 | 0.5 ± 0.0 | 0.0 ± 0.0 | 0.0 ± 0.0 | 0.0 ± 0.0 |
| 20:5n-3 | 0.2 ± 0.0 [a] | 0.9 ± 0.1 [b] | 0.7 ± 0.1 [b] | 0.0 ± 0.0 | 0.0 ± 0.0 | 0.0 ± 0.0 |
| 22:5n-3 | 0.6 ± 0.0 | 0.7 ± 0.0 | 0.7 ± 0.0 | 1.4 ± 0.1 | 1.4 ± 0.0 | 1.5 ± 0.1 |
| 22:6n-3 | 5.8 ± 0.4 [a] | 8.8 ± 0.3 [b] | 7.9 ± 0.4 [c] | 12.8 ± 0.3 [a] | 19.7 ± 0.5 [b] | 21.0 ± 0.3 [b] |
| **n-3** | **7.1 ± 0.4 [a]** | **11.0 ± 0.3 [b]** | **9.8 ± 0.4 [c]** | **14.2 ± 0.4 [a]** | **21.1 ± 0.5 [b]** | **22.5 ± 0.4 [b]** |
| **others** | **0.1 ± 0.0** | **0.1 ± 0.0** | **0.1 ± 0.0** | **0.0 ± 0.0** | **0.0 ± 0.0** | **0.0 ± 0.0** |
| μg/mg | 46.2 ± 2.0 | 44.3 ± 2.2 | 49.9 ± 4.3 | 19.8 ± 0.4 | 20.3 ± 0.5 | 20.3 ± 0.4 |

**Supplemental Table 3. The fatty acid profile of brain and eyes.**

Lipids were extracted by the Folch's method. The fatty acid profile was determined by GC-MS and expressed in mass %.

| % | Brain | | | Eyes | | |
|---|---|---|---|---|---|---|
| | Control | UN-DHA-O | EN-DHA-O | Control | UN-DHA-O | EN-DHA-O |
| 12:0 | 0.0 ± 0.0 | 0.0 ± 0.0 | 0.0 ± 0.0 | 0.0 ± 0.0 | 0.0 ± 0.0 | 0.1 ± 0.0 |
| 14:0 | 0.1 ± 0.0 | 0.1 ± 0.0 | 0.1 ± 0.0 | 1.0 ± 0.0 [a] | 1.1 ± 0.0 [ab] | 1.2 ± 0.0 [b] |
| 16:0 | 21.3 ± 0.3 | 20.9 ± 0.3 | 20.9 ± 0.3 | 18.7 ± 0.2 [a] | 21.0 ± 0.2 [b] | 21.4 ± 0.3 [b] |
| 18:0 | 19.7 ± 0.5 | 18.9 ± 0.4 | 18.7 ± 0.6 | 17.2 ± 0.4 | 16.4 ± 0.4 | 17.3 ± 0.8 |
| 20:0 | 0.1 ± 0.0 | 0.1 ± 0.0 | 0.1 ± 0.0 | 0.0 ± 0.0 | 0.0 ± 0.0 | 0.0 ± 0.0 |
| 22:0 | 0.1 ± 0.0 | 0.1 ± 0.0 | 0.1 ± 0.0 | 0.1 ± 0.0 | 0.0 ± 0.0 | 0.0 ± 0.0 |
| 24:0 | 0.0 ± 0.0 | 0.0 ± 0.0 | 0.0 ± 0.0 | 0.0 ± 0.0 | 0.0 ± 0.0 | 0.0 ± 0.0 |
| **saturated** | **41.3 ± 0.5** | **40.1 ± 0.5** | **39.9 ± 0.8** | **37.0 ± 0.5** [a] | **38.5 ± 0.4** [b] | **40.0 ± 0.8** [b] |
| 16:1n-7 | 0.3 ± 0.0 | 0.4 ± 0.0 | 0.4 ± 0.0 | 2.4 ± 0.2 | 2.5 ± 0.1 | 3.3 ± 0.3 |
| 18:1n-7 | 3.4 ± 0.1 | 3.4 ± 0.1 | 3.3 ± 0.1 | 3.3 ± 0.0 | 3.2 ± 0.0 | 3.2 ± 0.1 |
| 20:1n-7 | 0.2 ± 0.0 | 0.2 ± 0.0 | 0.2 ± 0.0 | 0.1 ± 0.0 | 0.0 ± 0.0 | 0.0 ± 0.0 |
| **n-7** | **3.9 ± 0.1** | **4.0 ± 0.1** | **3.9 ± 0.1** | **5.8 ± 0.2** | **5.7 ± 0.2** | **6.5 ± 0.4** |
| 16:1n-9 | 0.1 ± 0.0 | 0.1 ± 0.0 | 0.1 ± 0.0 | 0.3 ± 0.0 [a] | 0.2 ± 0.0 [b] | 0.2 ± 0.0 [b] |
| 18:1n-9 | 13.8 ± 0.3 | 15.0 ± 0.5 | 14.3 ± 0.4 | 13.2 ± 0.4 | 13.7 ± 0.3 | 14.1 ± 0.6 |
| 20:1n-9 | 0.6 ± 0.0 | 0.7 ± 0.1 | 0.7 ± 0.0 | 0.2 ± 0.0 [a] | 0.1 ± 0.0 [b] | 0.1 ± 0.0 [b] |
| 20:2n-9 | 0.0 ± 0.0 | 0.0 ± 0.0 | 0.0 ± 0.0 | 0.0 ± 0.0 | 0.0 ± 0.0 | 0.0 ± 0.0 |
| 20:3n-9 | 0.0 ± 0.0 | 0.0 ± 0.0 | 0.0 ± 0.0 | 0.2 ± 0.0 | 0.1 ± 0.0 | 0.1 ± 0.0 |
| 22:1n-9 | 0.1 ± 0.0 | 0.1 ± 0.0 | 0.1 ± 0.0 | 0.0 ± 0.0 | 0.0 ± 0.0 | 0.0 ± 0.0 |
| 24:1n-9 | 0.0 ± 0.0 | 0.0 ± 0.0 | 0.0 ± 0.0 | 0.0 ± 0.0 | 0.0 ± 0.0 | 0.0 ± 0.0 |
| **n-9** | **14.6 ± 0.3** | **15.9 ± 0.6** | **15.2 ± 0.4** | **13.9 ± 0.4** | **14.1 ± 0.3** | **14.5 ± 0.6** |
| 18:2n-6 | 0.4 ± 0.0 | 0.4 ± 0.0 | 0.4 ± 0.0 | 2.0 ± 0.1 | 1.9 ± 0.1 | 2.2 ± 0.2 |
| 18:3n-6 | 0.0 ± 0.0 | 0.0 ± 0.0 | 0.0 ± 0.0 | 0.0 ± 0.0 | 0.0 ± 0.0 | 0.0 ± 0.0 |
| 20:2n-6 | 0.0 ± 0.0 | 0.0 ± 0.0 | 0.1 ± 0.0 | 0.1 ± 0.0 [a] | 0.0 ± 0.0 [b] | 0.0 ± 0.0 [b] |
| 20:3n-6 | 0.2 ± 0.0 | 0.3 ± 0.0 | 0.3 ± 0.0 | 0.3 ± 0.0 [a] | 0.2 ± 0.0 [b] | 0.2 ± 0.0 [b] |
| 20:4n-6 | 12.7 ± 0.2 | 12.2 ± 0.2 | 12.3 ± 0.3 | 13.3 ± 0.2 [a] | 11.8 ± 0.2 [b] | 10.9 ± 0.2 [c] |
| 22:4n-6 | 3.3 ± 0.1 | 3.2 ± 0.1 | 3.2 ± 0.1 | 1.9 ± 0.0 [a] | 1.2 ± 0.0 [b] | 1.1 ± 0.0 [b] |
| 22:5n-6 | 0.7 ± 0.0 [a] | 0.5 ± 0.0 [b] | 0.5 ± 0.0 [b] | 0.5 ± 0.0 [a] | 0.1 ± 0.0 [b] | 0.1 ± 0.0 [b] |
| **n-6** | **17.3 ± 0.4** | **16.6 ± 0.3** | **16.8 ± 0.4** | **18.1 ± 0.2** [a] | **15.2 ± 0.2** [b] | **14.5 ± 0.3** [b] |
| 18:3n-3 | 0.0 ± 0.0 | 0.0 ± 0.0 | 0.0 ± 0.0 | 0.0 ± 0.0 | 0.0 ± 0.0 | 0.0 ± 0.0 |
| 20:5n-3 | 0.0 ± 0.0 | 0.0 ± 0.0 | 0.0 ± 0.0 | 0.0 ± 0.0 | 0.0 ± 0.0 | 0.0 ± 0.0 |
| 22:5n-3 | 0.1 ± 0.0 | 0.2 ± 0.0 | 0.2 ± 0.0 | 0.7 ± 0.0 | 0.7 ± 0.0 | 0.7 ± 0.0 |
| 22:6n-3 | 22.8 ± 0.3 | 23.2 ± 0.3 | 24.0 ± 0.5 | 24.4 ± 0.6 | 25.8 ± 0.3 | 23.8 ± 0.7 |
| **n-3** | **22.9 ± 0.3** | **23.4 ± 0.3** | **24.2 ± 0.5** | **25.1 ± 0.6** | **26.5 ± 0.3** | **24.5 ± 0.7** |
| **others** | **0.0 ± 0.0** | **0.0 ± 0.0** | **0.0 ± 0.0** | **0.1 ± 0.0** | **0.0 ± 0.0** | **0.0 ± 0.0** |
| µg/mg | 32.3 ± 0.9 | 33.1 ± 0.9 | 31.3 ± 0.8 | 4.1 ± 0.1 | 4.1 ± 0.1 | 3.9 ± 0.2 |
